# Skin-Derived Mesenchymal Stem Cells Help Restore Function to Ovaries in a Premature Ovarian Failure Mouse Model

**DOI:** 10.1371/journal.pone.0098749

**Published:** 2014-05-30

**Authors:** Dongmei Lai, Fangyuan Wang, Zhangli Dong, Qiuwan Zhang

**Affiliations:** The Center of Research Laboratory, and Department of Gynecology, The International Peace Maternity and Child Health Hospital, School of Medicine, Shanghai Jiaotong University, Shanghai, China; Imperial College London, United Kingdom

## Abstract

Skin-derived mesenchymal stem cells (SMSCs) can differentiate into the three embryonic germ layers. For this reason, they are considered a powerful tool for therapeutic cloning and offer new possibilities for tissue therapy. Recent studies showed that skin-derived stem cells can differentiate into cells expressing germ-cell specific markers *in vitro* and form oocytes *in vivo*. The idea that SMSCs may be suitable for the treatment of intractable diseases or traumatic tissue damage has attracted attention. To determine the ability of SMSCs to reactivate injured ovaries, a mouse model with ovaries damaged by busulfan and cyclophosphamide was developed and is described here. Female skin-derived mesenchymal stem cells (F-SMSCs) and male skin-derived mesenchymal stem cells (M-SMSCs) from red fluorescence protein (RFP) transgenic adult mice were used to investigate the restorative effects of SMSCs on ovarian function. Significant increases in total body weight and the weight of reproductive organs were observed in the treated animals. Both F-SMSCs and M-SMSCs were shown to be capable of partially restoring fertility in chemotherapy-treated females. Immunostaining with RFP and anti-Müllerian hormone (AMH) antibodies demonstrated that the grafted SMSCs survived, migrated to the recipient ovaries. After SMSCs were administered to the treated mice, real-time PCR showed that the expression levels of pro-inflammatory cytokines TNF-α, TGF-β, IL-8, IL-6, IL-1β, and IFNγ were significantly lower in the ovaries than in the untreated controls. Consistent with this observation, expression of oogenesis marker genes Nobox, Nanos3, and Lhx8 increased in ovaries of SMSCs-treated mice. These findings suggest that SMSCs may play a role within the ovarian follicle microenvironment in restoring the function of damaged ovaries and could be useful in reproductive health.

## Introduction

The skin is the largest organ of the body and has long been researched as a potential source of regenerative cells. For the past decade, several works have shown that skin-derived multipotent stem cells are capable of differentiating into cells from the three embryonic germ layers, including neural cells, adipocytes, insulin producing cells, chondrogenic cells, and osteogenic cells [1]–[5]. These findings indicate that skin can be a good alternative source of progenitor cells for the study of development and differentiation.

Recently, Dyce et al. reported that stem cells from fetal porcine skin can express germ cell markers and form oocyte-like cells *in vitro* [6]–[7]. They demonstrated that newborn mouse skin-derived stem cells are capable of differentiating into oocyte-like cells and forming oocytes after transplantation of cell aggregates into immunodeficient mice [8]. These findings suggest that skin-derived stem cells can differentiate into germ cells and this may be useful for anti-aging treatment.

Skin-derived mesenchymal stem cells (SMSCs) are primitive, unique, multipotent stem cells. Apart from their multilineage differentiation ability, SMSCs have inherent host compatibility, immunosuppressive ability, susceptibility to gene modification, and extensive capacity for *in vitro* expansion [9]–[10]. Here, female skin-derived mesenchymal stem cells (F-SMSCs) and male skin-derived mesenchymal stem cells (M-SMSCs) from red fluorescent protein (RFP) transgenic adult mice were used to determine the impact of cell transplantation on female reproductive function using a preclinical mouse model of chemotherapy-induced premature ovarian failure. The present work is the first to demonstrate that SMSCs can be grafted into the ovaries of chemotherapy-treated mice and restore ovarian function.

## Materials and Methods

### Isolation of SMSCs

RFP transgenic imprinting control region (ICR) mice were gifts from Professor Xizhi Guo [11]. Skin samples from the back of RFP transgenic 5-week-old male or female mice were carefully and separately dissected free from other tissue, placed in Hank’s balanced salt solution (HBSS), and cut into ≈1 mm^2^ pieces using dissecting scissors. Then, the segments were digested in 0.25% trypsin/EDTA at 37°C for 45 min. The resulting cell suspensions were seeded and cultured in a six-well plate in DMEM/F12 medium (Gibco, Invitrogen) containing 15% embryonic stem cell screened fetal bovine serum (ES-FBS; Gibco), 1% glutamine (Gibco), 1% penicillin/streptomycin (Gibco), and bFGF (Invitrogen 4 ng/mL) at 37°C in 5% CO_2_ in humidified air. Cells were passaged every 4–6 days.

### Flow Cytometry

The expression of CD73, CD14, CD34, and CD45 were evaluated on cells obtained from F-SMSCs or M-SMSCs. Cells (1×10^6^) were suspended in 2% BSA/PBS and labeled with PE rat anti-mouse CD73, PE rat anti-mouse CD14, FITC rat anti-mouse CD34, and FITC rat anti-mouse CD45 (all from BD, U.S.). Flow cytometry was performed using a FC500 flow cytometer (Beckman Coulter) and analyzed by Beckman Coulter CXP software.

### Differentiation of SMSCs

F-SMSCs or M-SMSCs were cultured in StemXVivo mesenchymal stem cell expansion media (R&D Systems) and differentiation was induced as indicated using the media supplements included in the mouse mesenchymal stem cell functional identification kit (R&D Systems). Markers of osteocyte and chondrocyte lineages were detected using a sheep anti-mouse osteocalcin polyclonal antibody and a sheep anti-mouse collagen II antigen affinity-purified polyclonal antibody, respectively. In addition, the frozen sections were prepared to do the Oil Red O for lipid staining (Sigma-Aldrich).

### Animals

A total of 105 six-week-old ICR female mice (non-transgenic) were sterilized by intraperitoneal injection of busulfan (30 mg/kg; resuspended in DMSO) and cyclophosphamide (120 mg/kg resuspended in DMSO) once and were observed for 1 week. The age-matched females injected with DMSO only were used as non-sterilized normal controls (n = 30). All animal procedures were approved by the Institutional Animal Care and Use Committee of Shanghai and were performed in accordance with the National Research Council Guide for Care and Use of Laboratory Animals.

### Transplantation of SMSCs, Total Body Weight Assessment, Tissue Preparation, and Mating Trials

Recipients were anesthetized by an intraperitoneal injection of pentobarbital sodium (45 mg/kg body weight). Approximately 20 µL of cell suspension containing 2×10^6^ F-SMSCs (n = 25) or M-SMSCs (n = 25), or 20 µL of culture medium (for the untreated control group; n = 15) were injected into the recipients via the tail vein 1 week after chemotherapy.

After intravenous injection of the SMSCs, treated and control animals were weighed. Animals were euthanized under anesthesia at 1, 2, 3, 4, 5, 6, 7, and 8 weeks after cell transplantation. Organ samples (brain, lung, heart, liver, spleen, femur, fat, ovary, uterus, vagina, and cervix) were collected, weighed, and stored at −80°C until further processing.

Treated (F-SMSC group, n = 13; M-SMSC group, n = 17) female, untreated female (n = 10), and normal control mice (n = 8) were housed with ICR males after chemotherapy for one month. Normal control mating trials using untreated mice were conducted. Adult males of proven fertility were housed with females at ratio of 1∶2. The number of offspring per litter was recorded.

Ovarian volumes were determined based on every 20th section in which the area of the ovarian tissue was obtained for each section (spaces and outside tissues were excluded) and the areas (mm^2^) were multiplied by the section intervals in mm. Values are expressed as µl (mm^3^) [12].

Follicle counts were made using a modification by Flaws et al. [13]. Slides were coded so the counter was blind to the genotype of the animal. Follicles from every 10th section from the paraffin series were categorized as to whether they were primordial, primary, preantral, or antral.

### Immunofluorescence Staining

Ovaries from recipient and control mice were fixed with the Tissue-Tek OCT Compound (Sakura Finetek Middle East, Dubai, United Arab Emirates) and sliced in 5 µm thick sections. Slides were washed twice with PBS and kept in blocking solution for 30 min at room temperature. Slides were then incubated with rabbit polyclonal anti-RFP (1∶200; Abcam) or anti-AMH (1∶200, AbD SeroTec) at 4°C overnight. Non-immune immunoglobulins of the same isotype as the primary antibody were used as negative controls. Cells or sections were washed three times with 1×PBS and probed with TRITC–labeled IgG (1∶200, Santa Cruz, CA, U.S.) or FITC-labeled IgG (1∶200, Santa Cruz, CA, U.S.). Fluorescence images were taken using a Leica DMI3000 microscope (Wetzlar, Germany).

### RNA Extraction and Real-time qPCR Analysis

Total RNA was extracted from snap-frozen ovaries using the RNeasy Mini Kit (Qiagne, Valencia, U.S.). Five hundred nanograms of total RNA from each sample was utilized for reverse transcription using the iScript cDNA Synthesis Kit (Bio-Rad, Hercules, U.S.). Real-time PCR was carried out on cDNA using IQ SYBR Green (Bio-Rad) with a Mastercycler ep realplex real-time PCR system (Eppendorf, Hamburg, Germany). All reactions were performed using a 25 µL volume. The primers for the marker genes are provided in Table 1. PCR was accomplished by an initial denaturation at 95°C for 5 min, followed by 40 cycles for 30 s at 95°C, 30 s at 60°C, and 30 s at 72°C. PCR without the template DNA was used as a negative control. The threshold cycle (Ct) values of each sample were used in the post-PCR data analysis. Normalization of mRNA levels was done using 18S RNA as an internal control.

**Table 1:** PCR primer sequences.

Gene product	Forward(F) and reverse(R) primers(5′ → 3′)	Size(bp)
TNF-α	F: GCCTCTTCTCATTCCTGCTT	203
	R: CACTTGGTGGTTTGCTACGA	
TGF-β	F: AGGGCTACCATGCCAACTTC	168
	R: CCACGTAGTAGACGATGGGC	
IL-6	F: CGGCCTTCCCTACTTCACAA	187
	R: TCTGCAAGTGCATCATCGTT	
IL-8	F: CCTAGGCATCTTCGTCCGTC	201
	R: TTCACCCATGGAGCATCAGG	
IL-l0	F: CCAAGCCTTATCGGAAATGA	162
	R: TTTTCACAGGGGAGAAATCG	
IFN-γ	F: GCGTCATTGAATCACACCTG	129
	R: TGAGCTCATTGAATGCTTGG	
IL-1β	F: ACCTCACAAGCAGAGCACAA	200
	R: TTGGCCGAGGACTAAGGAGT	
Nobox	F: GGCACTAGTATCGCCTCACC	186
	R: CATTGAGCTTGGGATGGGGT	
Nano3	F: CCGTGCCATCTATCAGTCCC	179
	R: AATTCCGGGTGGTGTAGCAG	
Lhx8	F: GCCCTTGTTACCCCATCCAA	215
	R: AGTGTCTTGCAGGCCAACTC	
18S	F: GTAACCCGTTGAACCCCATT	202
	R: CCATCCAATCGGTAGTAGCG	

### Statistical Analysis

Relative gene expression means were compared by ANOVA using Microsoft Excel software. Offspring distribution was assayed using Kruskal-Wallis test. Differences were considered statistically significant when *P*<0.05.

## Results

### Characteristics of SMSCs from RFP Transgenic Mice

**Figure 1:**
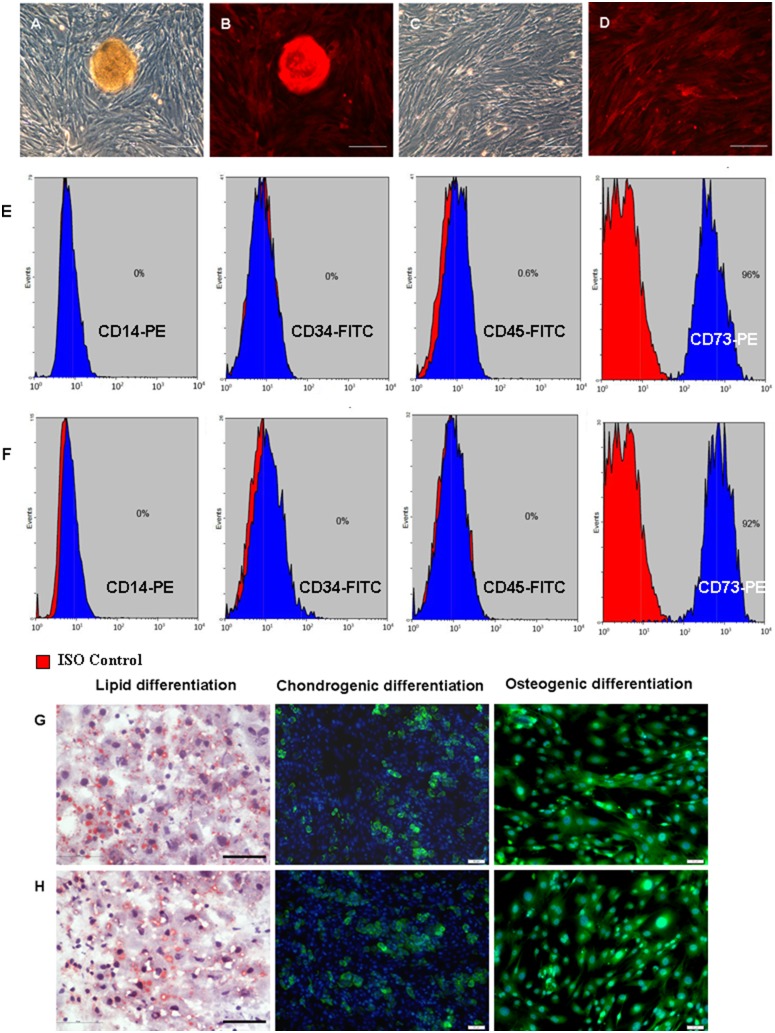
The characterization and differentiation of mouse SMSCs. Morphology of A–B) F-SMSCs (a cell cluster in the middle of the dish) and C–D) M-SMSCs from RFP transgenic mice (scale bar, 200 µm). E) Phenotype of F-SMSCs by flow cytometry. Of the F-SMSCs, 96% expressed CD73 but not CD45, CD34, or CD14. F) M-SMSCs detected by flow cytometry. Of the M-SMSCs, 92% expressed CD73 but not CD45, CD34, or CD14. G) F-SMSCs and H) M-SMSCs were able to differentiate into adipocytes (oil red O staining), chondroblasts, and osteoblasts under standard in vitro differentiation conditions. The nuclei were counterstained with DAPI (blue) (scale bar, 50 µm).

The cells presumed to be F-SMSCs and M-SMSCs were derived from female and male RFP transgenic ICR mice. The 5th passage of F-SMSCs and M-SMSCs had similar morphology. Most of the cells were spindle-shaped and similar to fibroblast-like cells (Fig. 1A–D). The cells exhibited a rapid growth with cell clustering (Fig. 1A–B).

Flow cytometry was used to detect the phenotype of the 5th passage F-SMSCs and M-SMSCs. The results showed that 96% cells of F-SMSCs or 92% cells of M-SMSCs were CD73-positive and lack of expression of CD14, CD34 and CD45 (Fig. 1E–F).

Further, both of F-SMSCs and M-SMSCs were multipotent, as determined by their ability to differentiate into adipocytes, chondroblasts, and osteoblasts (Fig. 1G&H).

These results showed that mouse skin-derived cells had the characteristics of mesenchymal stem cells.

### SMSC Transplantation Improved the Total Body and Reproductive Organ Weights in Sterilized Mice

**Figure 2:**
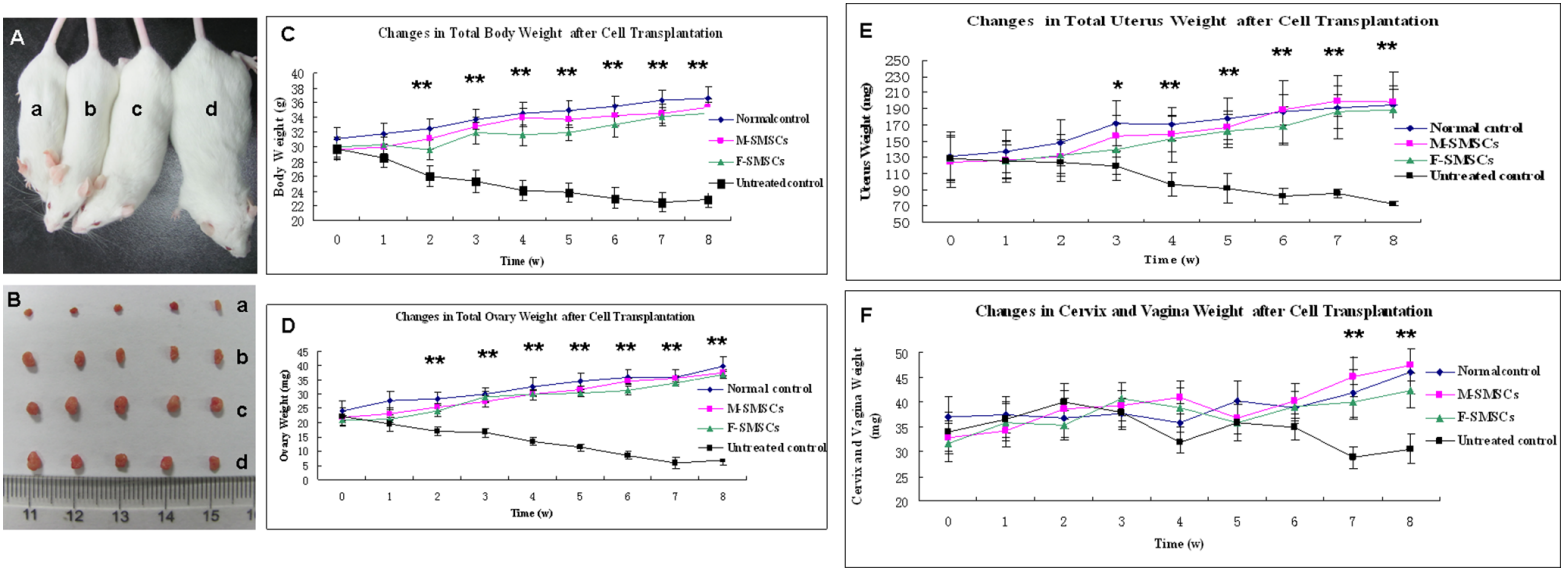
Changes in total body weight and weight of reproductive organs in treated, untreated control, and normal control animals over 8 weeks. A) Representative photograph depicting the body type of 12 week-old mice in the (a) untreated control group, treated groups (b, F-SMSCs; c, M-SMSCs), and (d) normal control group. B) The total body weight of untreated mice decreased over the study period. However, mice in both the F-SMSC-treated and M-SMSC-treated groups weighed significantly more than mice in the untreated control group from the second week onward (**, *P*<0.01). C) Representative photograph of ovaries removed from (a) the untreated control group, treated groups (b, F-SMSCs; c, M-SMSCs), and (d) normal control group. D) As indicated, the total weight of ovaries in the treated groups (both M-SMSC- and F-SMSC-treated) showed remarkable increases over the study period except before the first week (**, *P*<0.01). E) The total weight of the uteruses in the treated group was significantly higher than that of untreated controls from the third week onward (*, *P*<0.05; **, *P*<0.01). F) There was no obvious change in the total weight of the cervixes and vaginas during the study period until after the 7th and 8th weeks (**, *P*<0.01).

To evaluate the effect of SMSCs on the total body and reproductive organ weights in chemotherapy sterilized mice, F-SMSCs and M-SMSCs were separately transplanted by tail vain. Significant greater total body weight were observed in both F-SMSC- and M-SMSC-treated animals than in untreated control animals at each point in time after cell transplantation up to 2 weeks (*P*<0.01, Fig. 2A–B). Ovaries were weighed at each point in time at which animals were killed. As with the total body weight, statistical analysis revealed that the weight of the ovaries increased in treated vs. untreated control animals at each time point after cell transplantation up to 2 weeks (*P*<0.01, Fig. 2C–D). Reproductive organs, including the uterus, vagina, and cervix, which are highly modulated, both structurally and functionally, by estrogen, were also weighed. Results showed a marked increase in uterus weight at each point in time after cell transplantation up to 4 weeks (*P*<0.01, Fig. 2E). Statistically significant differences were observed in vagina and cervix weights for treated vs. untreated control animals after 7 weeks (*P*<0.01, Fig. 2F).

We did not see any signs of graft versus host disease (GVHD) in any transplanted mice. This was because the transplanted cells were derived from ICR mice, and all ICR mice used in this study were immunologically compatible.

### SMSC Transplantation Re-established Fertility in Sterilized Mice

**Figure 3:**
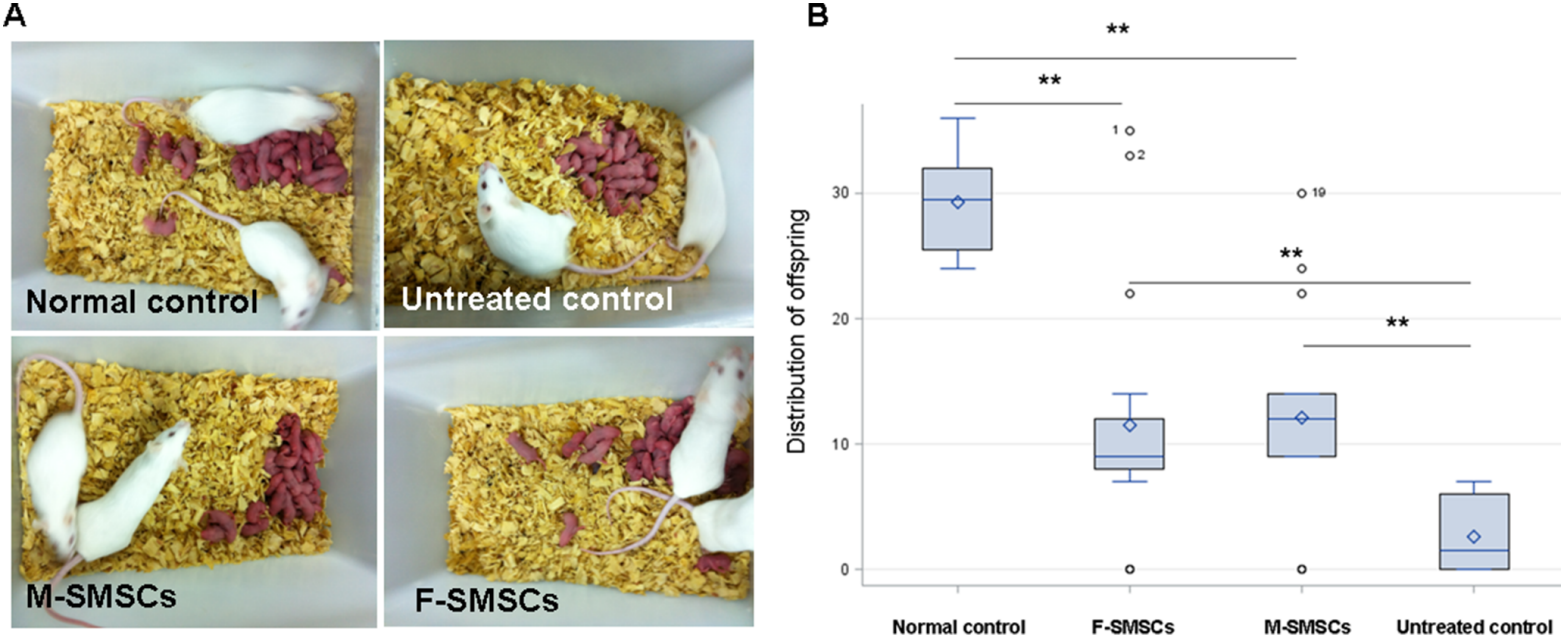
Offspring produced by mating after SMSC transplantation into the mice sterilized by chemotherapy and those produced by untreated controls and normal controls. A) Litters of the different groups. B) Distribution of offspring in the treated and untreated groups. On average, significantly more pups were born to the mice that received chemotherapy plus F-SMSC- or M-SMSC-transplantation than to untreated controls (**, *P*<0.01). However, the average number of pups produced by the normal control mice was still higher than the average number of pups from mice receiving F-SMSC- or M-SMSC-transplantation (**, *P*<0.01).

Over the course of the 3 month mating trial, sterilized female mice that did not receive SMSC transplantation became infertile until after a two-month recovery period. Half (5 of 10 mice) achieved only one pregnancy with 26 offspring, including 6 live births and 20 stillbirths. In mice that underwent M-SMSC or F-SMSC transplantation 1 week after chemotherapy, 82.4% (14 of 17) of the mice achieved at least one pregnancy, producing 135 living offspring. In mice that underwent F-SMSC transplantation 1 week after chemotherapy, 76.9% (10 of 13) of the mice achieved at least one pregnancy, producing 114 living offspring. Among M-SMSC mice, 17.6% (3 of 17) achieved two pregnancies, producing 32 living offspring in the second pregnancy. Among F-SMSC mice, 23.1% (3 of 13) achieved two pregnancies, producing 26 living offspring in the second pregnancy. Among M-SMSC mice, 11.8% (2 of 17) achieved three pregnancies, producing 26 living offspring in the third pregnancy. Among F-SMSC mice, 15.4% (2 of 13) achieved three pregnancies, producing 18 living offspring in the third pregnancy. None of the untreated controls had more than one litter (Fig. 3B, *P*<0.001). On average, significantly more pups were produced by mice receiving chemotherapy plus M-SMSC or F-SMSC transplantation (9.5±4.9 or 8.8±4.6 pups per mouse, respectively) than by untreated controls (2.8±2.7 pups per mouse; *P*<0.01).

Normal animals without chemotherapy were also used as controls (n = 8), with 100% (8 of 8) achieving live births in three pregnancies during the 3-month mating trial. Treated mice had significantly fewer pregnancies (Table 2, *P*<0.01). The average number of pups from each normal control mouse was 29.8±5.6, and the average number of pups from each mouse receiving M-SMSC or F-SMSC transplantation was 9.5±4.9 or 8.8±4.6, respectively (Table 2, *P*<0.01).

**Table 2:** Summary of female fertility study.

	Normal control	M-SMSCs transplantation	F-SMSCs transplantation	Non-treated control
No. of Animal (female)	8	17	13	10
No. evaluated	8	17	13	10
No. Died or Sacrificed Moribund	0	0	0	0
No. animals mated	8	17	13	10
No. pregnant Animals	8	14	10	5
No. of Live embryo	234	193	158	6
Mean No. embryo (Mean ± S.D.)	29.8±5.6**	8.8±4.6	9.5±4.9	2.8±2.7**
No. of Dead embryo	0	0	0	20
Mean No. Dead embryo (Mean ± S.D.)	0	0	0	2.5±2.2**
Number of pregnancy (%)				
Up to 3 pregnancies (%)	100% (8/8)**	11.8% (2/17)	15.4% (2/13)	0**
Up to 2 pregnancies (%)	100% (8/8)**	17.6% (3/17)	23.1% (3/13)	0**
Up to 1 pregnancy (%)	100% (8/8)	82.4% (14/17)	76.9% (10/13)	50% (5/5)*

*P<0.01,

**P<0.05.

### These Results Showed that Ovarian Functions in Sterilized Female Mice were Partially Restored by SMSC Transplantation

SMSCs survived and infiltrated into the chemically-damaged murine ovarian tissue.

**Figure 4:**
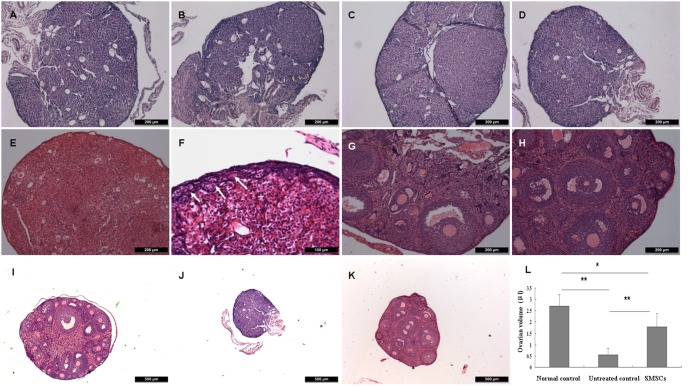
Histological evaluations of ovaries in treated, untreated control, and normal control animals by midline section and HE staining. Ovary sections from sterilized non-transplanted mice after (A) a 7-day recovery period and (B) a 14-day recovery period, which showing the hollow structure destroyed by chemotherapy. Ovary sections from sterilized non-transplanted mice showing fibrosis and reduced hollow structure after a (C) 28-day recovery period and (D) 2-month recovery period. Ovary sections from sterilized recipient mice at (E) 7 days, (F) 14 days, (G) 28 days, and (H) 2 months after transplantation of SMSCs. Primordial follicles are visible in F (arrows), developing follicles at various stages are visible in G and H. However, there were fewer such developing follicles in G than in H. The morphology of ovary section of normal (I), untreated (J), and treated (K) control animals. (H) The changes in the ovarian volumes in treated, untreated control, and normal control (**P<0.01, *P<0.05). Scale bars: (F) 100 µm, (A–E, G–H) 200 µm, (I–K) 500 µm.

Histological evaluations of untreated control ovaries were evaluated 7 days to 2 months after chemotherapy. Results showed that the chemotherapy regimen gradually destroyed total immature (primordial, primary, secondary) follicles with vacuolar structure remaining and fibrosis (Fig. 4A–D). However, the ovaries of mice receiving F-SMSC and M-SMSC transplantation possessed primordial, primary and developing follicles 14 days to 2 months after SMSC transplantation (Fig. 4F–H). Varied staged follicles in different groups were counted which showed that the SMSCs improved the number of primordial, primary and secondary follicels in treated ovaries (Fig. S1). Besides the changes of morphology of ovaries, the ovary volume declined 2 months after chemotherapy. Ovary volume in SMSC-treated mice increased significantly, but they were still smaller than normal control ovaries (Fig. 4I–L).

**Figure 5:**
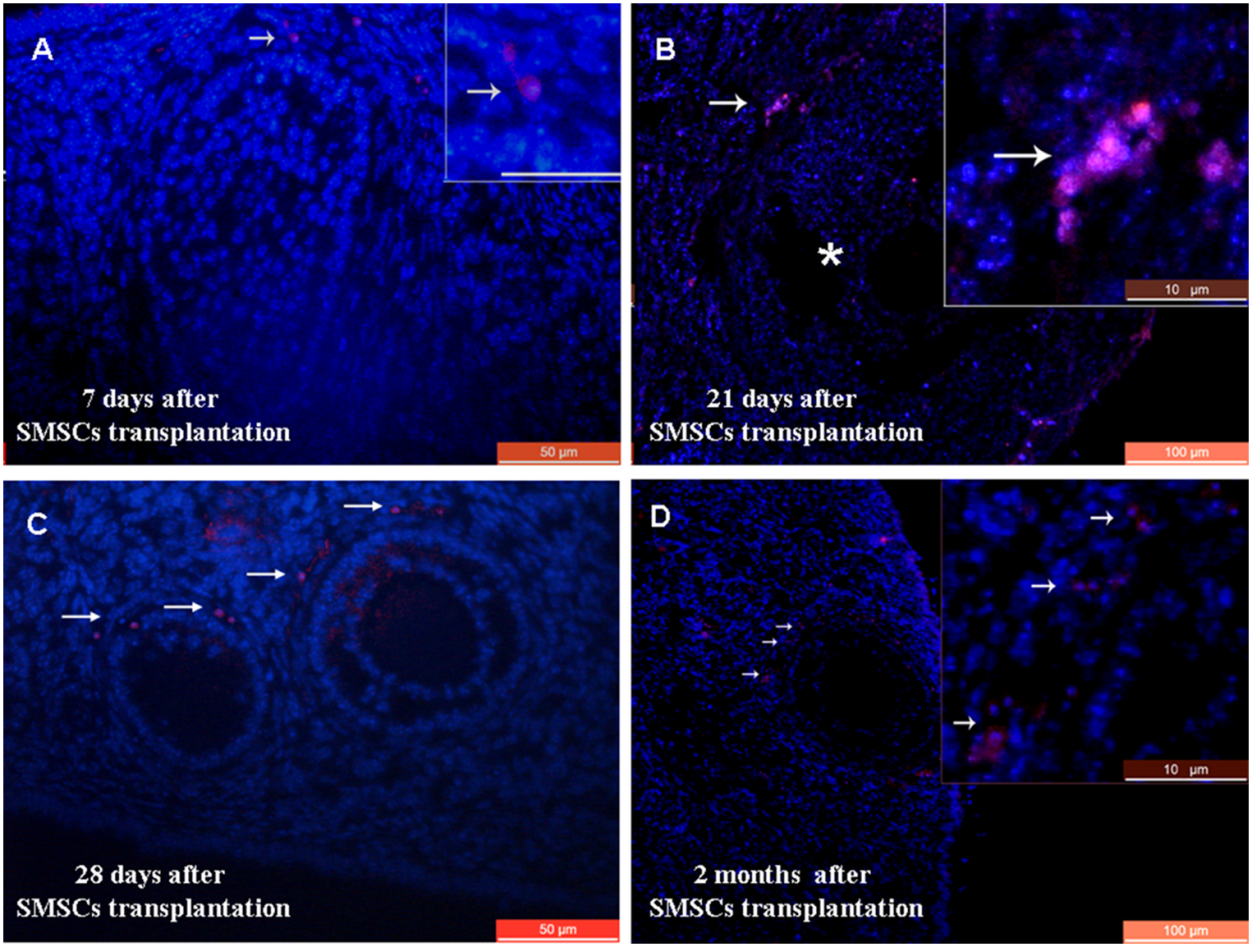
Transplantation of a line of RFP-transgenic SMSCs into chemotherapy-sterilized recipient mice. A) Immunofluorescence of ovarian sections from sterilized mice 7 days after transplantation with RFP-transgenic SMSCs. Arrows indicate RFP staining in the ovarian stroma. B) RFP staining was observed around the antral follicle 21 days after transplantation. Asterisks indicate the antral follicle. C) RFP-positive cells were observed around granulosa cells in recipient ovaries 28 days after SMSC transplantation. D) RFP staining was observed in antral follicles of recipient ovaries 2 months after SMSC transplantation. Scale bars: A, C) 50 µm; B, D) 100 µm; A, insets) 20 µm; and B, D insets) 10 µm.

Because SMSC transplantation improved the ovarian function of sterilized mice, we hypothesized that F-SMSCs or M-SMSCs had migrated and integrated in the damaged ovarian tissue. Indeed, SMSCs exhibited various degrees of distribution. At 7 days, 21 days, 28 days, and 2 months post-transplantation, cells were found in the stroma of the ovarian tissue (Fig. 5A–D).

**Figure 6:**
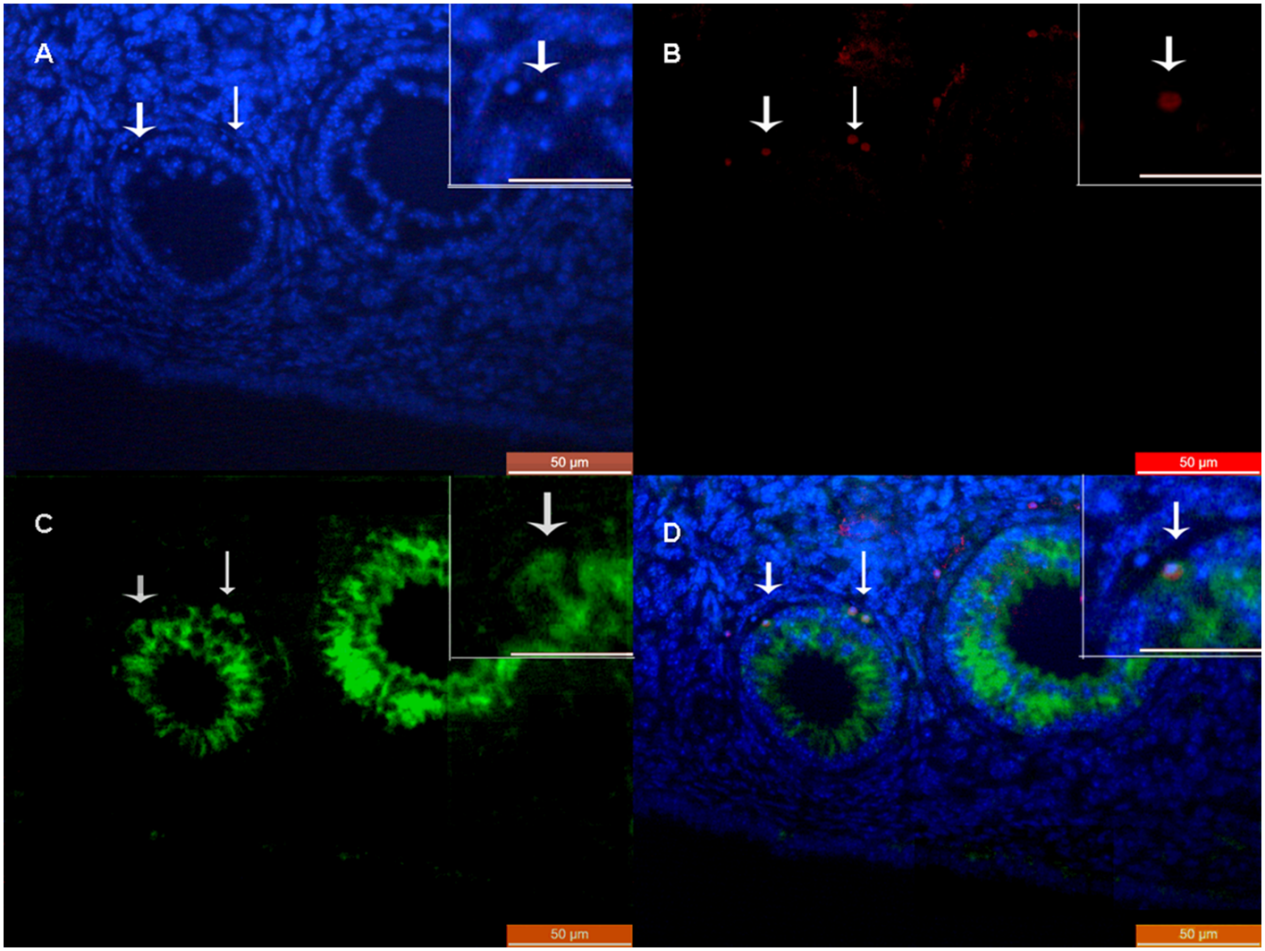
Double-staining of RFP and AMH in antral follicles of recipient ovaries 2 months after SMSC transplantation. A) DAPI. B) RFP staining cells. C) AMH staining cells. D) Merged image of A, B, and C. Scale bars: 50 µm; insets) 20 µm.

Anti-Müllerian hormone (AMH) expression is highest in granulosa cells of preantral and small antral follicles, and gradually diminishes in the subsequent stages of follicle development. AMH expression disappears when follicles become atretic. In this way, AMH was found to be a marker of granulosa cells [14]. At 2 months post-transplantation, accompanied by the development of follicles, the RFP staining cells were found in the granulosa cells around the oocytes, and some cells were co-stained with AMH (Fig. 6D).

Interestingly, no RFP signal was detected in other organs, including liver, kidneys, spleen, heart, lungs, brain, and bone marrow (BM) at 28 days post-transplantation. It is worth noting that 351 newborns produced by SMSC-treated mice were also RFP-negative by DNA extraction (data not shown).

### SMSC Administration Decreased Ovary Inflammation Induced by Chemotherapy and Improved Transcriptional Regulation of Early Folliculogenesis

Various chemotherapeutic agents have different gonadotoxic effects that may involve a variety of pathophysiological mechanisms, which need to be further elucidated. Evidence has shown that alkylating agents and cyclophosphamide therapy can cause follicular depletion and dose-dependent, direct destruction of oocytes. Studies have also found that cortical fibrosis and blood-vessel damage can be caused by alkylating agents [15]–[17]. In this way, inflammation may occur and be associated with chemotherapy damage to the ovaries.

**Figure 7:**
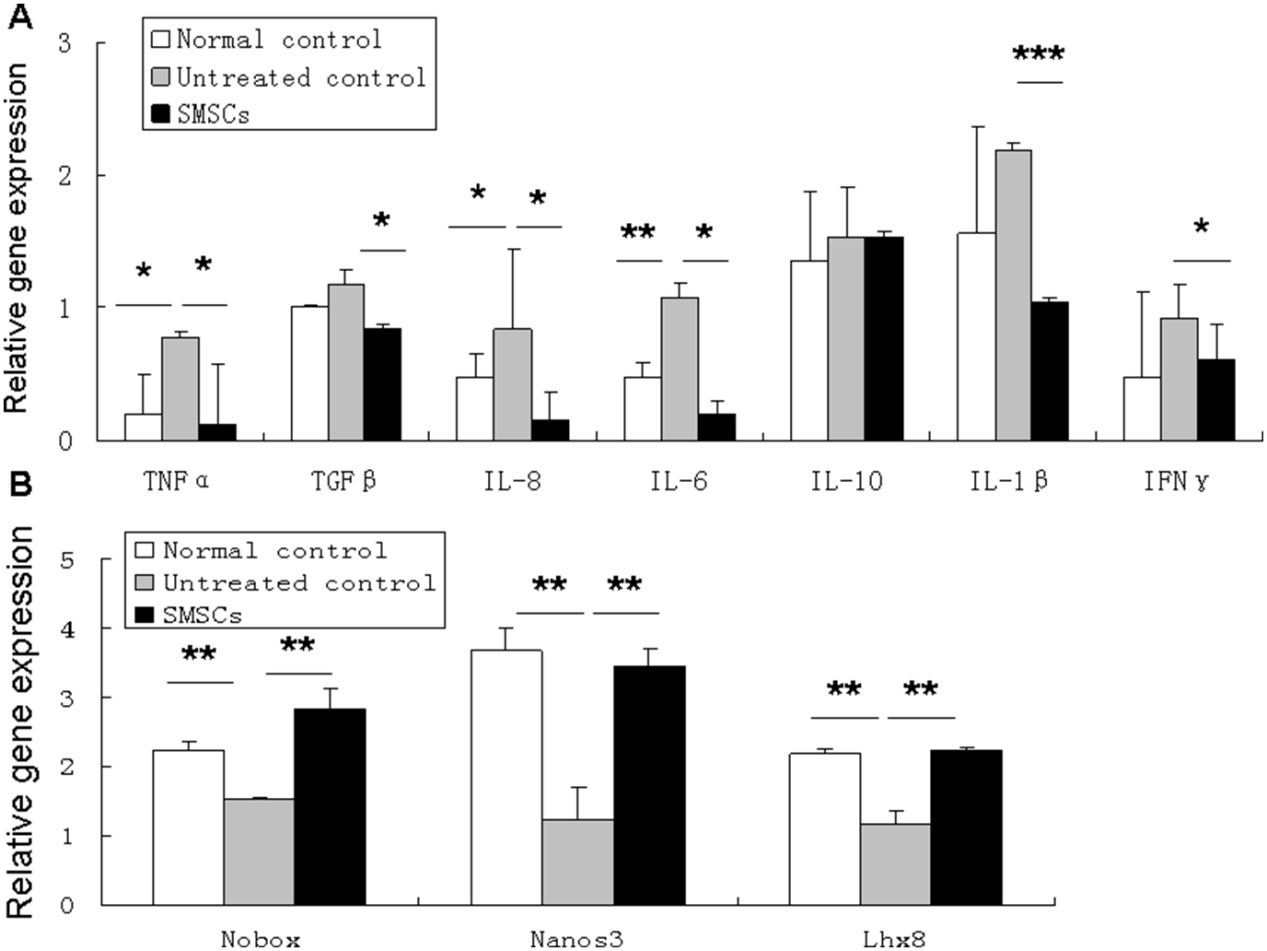
SMSC administration decreased ovary inflammation and improved folliculogenesis transcription. A) Analysis of pro- and anti-inflammatory cytokine gene expression as performed by qPCR. Administration of SMSCs 7 days after chemotherapy resulted in significantly less gene expression for TNF-α, TGF-β, IL-8, IL-6, IL-1β, and IFN-γ than in untreated control mice (*P<0.05, **P<0.01, ***P<0.01). B) Expression of oocyte-specific genes in treated and untreated recipient mouse ovaries. Quantitative real-time RT-PCR showed that the relative expression of Nobox, Nanos3, and Lhx 8 was significantly higher in the SMSC group compared than among untreated controls (**P<0.01). 18s rRNA served as an internal housekeeping gene. Data represent mean±SE of 3 independent experiments.

To determine the effect of SMSC administration on chemotherapy-induced inflammation of the ovary, qPCR was performed on day 7 after SMSC transplantation using probes specific for a number of pro- and anti-inflammatory cytokines. Gene expression was measured as the fold change relative to 18S. The mice that received chemotherapy had significantly more gene expression of several pro-inflammatory cytokines, such as TNF-α, IL-8, and IL-6 than normal control animals (Fig. 6A). The administration of SMSCs 7 days after chemotherapy was associated with significantly lower gene expression of TNF-α, TGF-β, IL-8, IL-6, IL-1β, and IFN-γ than in untreated animals (Fig. 7A). No significant differences between groups were detected for expression of anti-inflammatory cytokine IL-10 (Fig. 7).

To understand how SMSC administration acts on the transcriptional level in oogenesis, the gene expression of oocyte-specific transcriptional factors Nobox, Nanos3, and Lhx8 was detected in mouse ovaries. Newborn ovary homeobox (Nobox_ gene expression in the ovary is oocyte-specific and it is critical to in early folliculogenesis. Nanos3 expression results in a RNA binding protein whose role in germ cell development is highly conserved. The Lhx8 (LIM homeobox 8) gene expression, which occurs mainly in germ cells, results in a LIM-homeobox transcription factor that is critical to mammalian oogenesis [18]–[20]. Interestingly, on day 7 after SMSC transplantation, the expression of these genes was found to be significantly higher in the ovaries of recipient mice than in untreated control ovaries of sterilized mice (Fig. 7).

## Discussion

In the present study, the effects of SMSC transplantation on ovarian function in female mice with ovarian failure were evaluated. Administration of chemotherapy decreased not only the reproductive capacity but also the total body weight and reproductive organ weight of the mice. The transplantation of female or male SMSCs into the recipient mice strikingly improved both the body condition and ovarian function, as damaged by chemotherapy. This was consistent with previous reports [21]–[23]. The number of litters obtained by natural mating was significantly higher in mice treated with F-SMSCs and M-SMSCs compared with mice of the non-transplanted group. However, this difference was not found to completely match the fertility associated with mice prior to chemotherapy-induced injury.

When analyzing the distribution of transplanted mesenchymal stem cells, one group reported that transplantation of bone marrow cells harvested from TgOG2 transgenic donor females expressing GFP generated immature GFP-positive oocytes in sterilized female mice [22]. However, some studies found that bone marrow cells did not contribute to the formation of mature, ovulated oocytes. These cells, instead, migrated to the ovary via blood circulation and exhibited a blood leukocyte-like phenotype [24]. Recently, it has also been shown that new fertilizable oocytes could not be obtained from transplanted bone marrow cells in a mouse model treated with chemotherapeutic agents or with bovine embryonic ovarian tissue grafts [21].

This is the first report to show that RFP transgenic cells were detected in the stroma of recipient ovaries, mostly in the granulosa cells around the oocytes. AMH was used as the cell marker for granulosa cells because the members of the transforming growth factor β superfamily are highly expressed in granulosa cells of the preantral and small antral follicles in the ovary [14], [25]. Double staining for RFP and AMH was performed to assess the survival and differentiation of the transplanted SMSCs. Because AMH is a secreted protein, it was not possible to distinguish the co-localization of RFP and AMH well. However, some of the transplanted RFP-positive SMSCs may have entered the stroma, but not differentiated to germ cells. Previous studies have shown that apoptosis, the most prevalent mechanism behind oocyte loss, can be induced by chemotherapy in the granulosa cells of developing ovarian follicles [26]–[28]. The present results suggest that some of the transplanted SMSCs restored ovarian function by repairing cells damaged by chemotherapeutic agents. These findings further support the conclusion that normal cell-cell communication is critical to oocyte growth [29]–[31].

All the recipient ovarian sections were checked after SMSC transplantation, and only 20% of recipient ovaries were found to be positive for RFP (data not shown). This indicated that there is more than one mechanism of SMSC repairing ovarian function. For this reason, the ability of SMSCs to abrogate chemotherapy-induced ovary damage was explored next.

Recently, Park et al. investigated the apoptosis of ovarian cells in busulfan and cyclophosphamide (B/C)-treated mice. They showed that the apoptotic signaling mechanisms mediated by p53, FAS/FASL, and TNF signaling pathways were not involved in the depletion of female germ cells [26]. It is here suggested that other events, such as inflammation, may have occurred in the ovaries of B/C-treated mice. This showed that the expression of several pro-inflammatory cytokines, such as TNF-α, IL-8, and IL-6 increased in the damaged ovaries. TNF-α, IL-8, IL-6, IL-1β, and IFN-γ were significantly lower after SMSC transplantation than in untreated mice. In this way, SMSC transplantation can mitigate inflammatory cytokine activity and modulate the inflammatory response in the ovaries. Some follicular marker genes, such as Nobox, Nanos3, and Lhx8, are known to be involved in the development of primordial follicles and might be impaired by B/C treatment [26]. The current results showed that SMSC transplantation up-regulated oocyte-specific transcriptional factors, including Nobox, Nanos3, and Lhx8, in recipient mouse ovaries that had been damaged by chemotherapy. In this way, results suggested that part of the effect of SMSC transplantation is the reduction of damage to the ovary produced by chemotherapy. This effect is likely mediated by their anti-inflammatory properties, which may further facilitate oogenesis and ultimately restore ovarian function.

It is here proposed that SMSCs function primarily by reactivating host oogenesis. Chemotherapy damages ovaries through either direct effects of the drugs on apoptosis of granulosa cells or indirect effects of the drugs on the microenvironments that support the development of oocytes. It is possible that the transplanted somatic cells or factors released from the transplanted cells repaired the gonadal microenvironment and improved the conditions for development of germ cells. In this way, SMSCs might differentiate into stroma cells, reduce the inflammatory response in the ovaries, and improve the germ cell niche, which is important for folliculogenesis and fertility.

First reported by Peterson et al. in 1999, bone marrow from male rats was transplanted into lethally irradiated syngeneic females in order to trace the donor cells in the recipients by using Y chromosome-specific DNA probes without causing immune rejection [32]. Takehara et al. recently described the effectiveness of male adipose-derived mesenchymal stem cells after their transplantation into ovaries and found these cells to have a role in restoring damaged ovarian function [33]. Consistent with these reports, male derived SMSC were found to have the same restorative effects on ovarian function as female derived SMSCs and that they do not have a higher rate of immune rejection.

### Conclusion

SMSCs can affect chemotherapy-induced premature ovarian failure and rescue fertility in sterilized mice. The mechanisms not only involve the migration and engraftment of SMSCs in host ovaries but are also likely related to the anti-inflammatory properties of the cells. This study is the first to demonstrate the efficacy of SMSCs in preserving and restoring ovarian function in a premature ovarian failure mouse model. The present findings have implications for the future use of SMSCs in improving the quality of life in cancer survivors.

## Supporting Information

Figure S1 **SMSCs improved the number of primordial, primary, and secondary follicles in treated ovaries.** Follicle counts of primordial, primary, secondary, and atretic follicles in ovaries of each group, including normal controls, untreated controls, and SMSC-treated animals.
(TIF)
