# Supplementary figures and images for: Skin-Derived Mesenchymal Stem Cells Help Restore Function to Ovaries in a Premature Ovarian Failure Mouse Model
Source: PLoS One. 2014 May 30;9(5):e98749. doi: 10.1371/journal.pone.0098749 (PMC4039525; doi:10.1371/journal.pone.0098749)

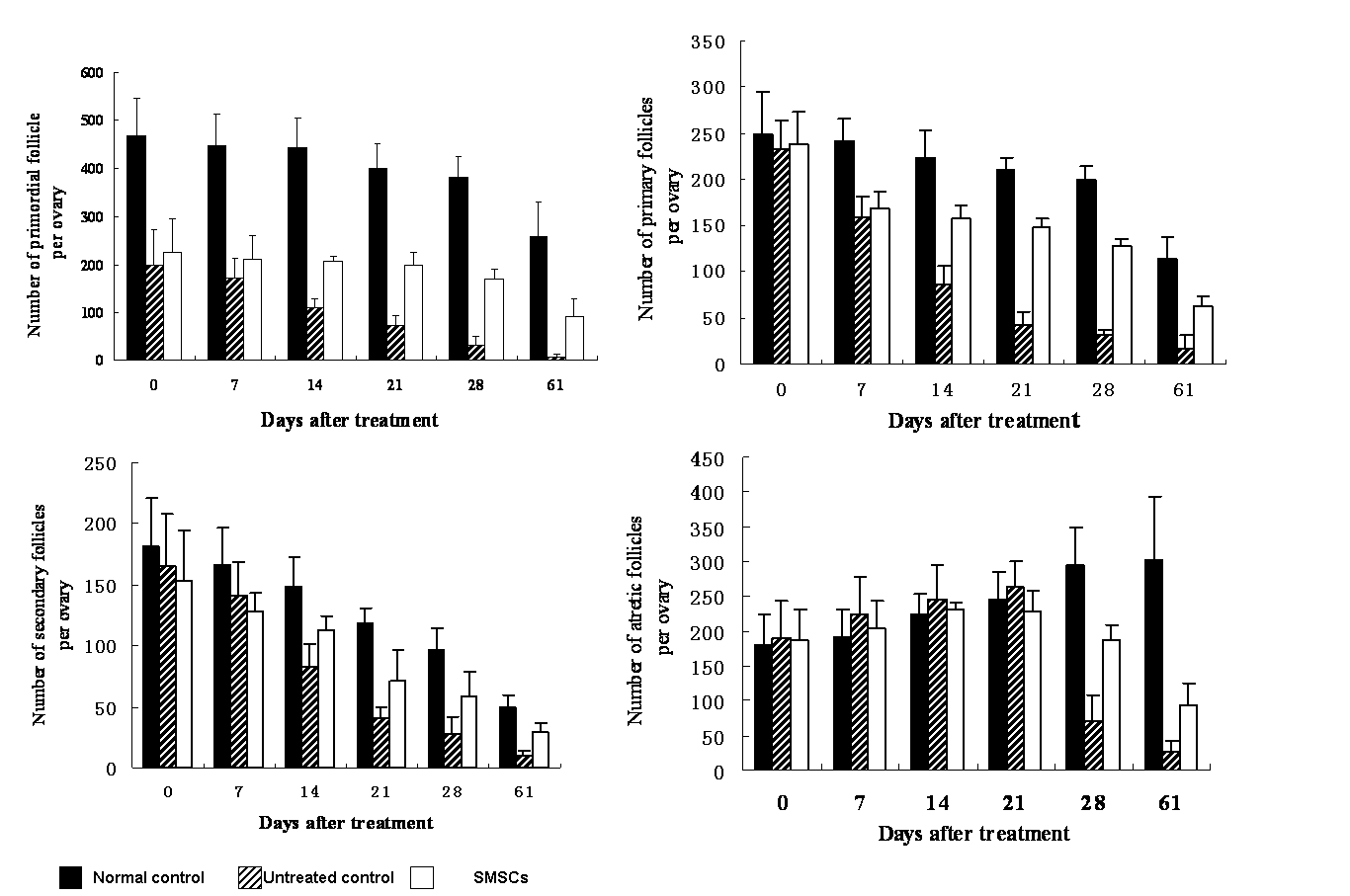

Supplement: Figure S1 — SMSCs improved the number of primordial, primary, and secondary follicles in treated ovaries. Follicle counts of primordial, primary, secondary, and atretic follicles in ovaries of each group, including normal controls, untreated controls, and SMSC-treated animals. (TIF) [file pone.0098749.s001.tif]
